# Effect of environmental temperature on oxidative status, inflammatory response and endocannabinoid system at oestrus in dairy cows

**DOI:** 10.1002/vro2.70037

**Published:** 2026-05-24

**Authors:** Alice Carbonari, Lorenza Frattina, Matteo Burgio, Vincenzo Cicirelli, Michal Andrzej Kosior, Alessandra Sfacteria, Bianca Gasparrini, Gabriele Marino, Roberta Verde, Fabiana Piscitelli, Annalisa Rizzo

**Affiliations:** ^1^ Department of Veterinary Medicine University of Bari Aldo Moro Valenzano Italy; ^2^ Department of Veterinary Medicine and Animal Production Federico II University of Naples Naples Italy; ^3^ Department of Veterinary Sciences University of Messina Messina Italy; ^4^ Endocannabinoid Research Group Institute of Biomolecular Chemistry—National Research Council Pozzuoli Italy

**Keywords:** dairy cattle, endocannabinoid system, heat stress, inflammatory cytokines, oxidative status, seasonal variation

## Abstract

**Background:**

Heat stress (HS) poses a significant challenge to dairy production under climate change, as it compromises fertility, productivity and overall health in cows. This seasonal observational cross‐sectional study investigated how environmental temperature affects oxidative status, inflammatory responses and endocannabinoid system (ECS) in dairy cows, comparing winter and summer conditions.

**Methods:**

Sixty Italian Holstein cows, 20 per farm, were enrolled from three farms in southern Italy. In winter and summer, temperature‒humidity index (THI) was recorded using data loggers and blood samples were collected from each cow's coccygeal vein during oestrus. Serum samples were analysed to evaluate the oxidative status as reactive oxygen metabolites (d‐ROMs) and biological antioxidant potential (BAP), inflammatory responses as tumour necrosis factor‐alpha (TNF‐α) and interleukin (IL)‐10, and ECS as anandamide (AEA) and 2‐arachidonoylglycerol (2‐AG).

**Results:**

Temperature‒humidity index was significantly higher in summer than in winter (*p* < 0.001), confirming HS exposure. Anandamide levels showed no significant seasonal variation, while 2‐AG concentrations were significantly reduced in summer compared to winter (*p* < 0.01). Oxidative stress increased in summer, with lower BAP (*p* < 0.05) and higher d‐ROMs (*p* < 0.001). Tumour necrosis factor‐alpha and IL‐10 were significantly higher in summer than in winter (*p* < 0.001 and <0.05).

**Conclusion:**

The study demonstrates that summer HS could alter oxidative balance, stimulates inflammatory pathways and modulates ECS activity in dairy cows. Reduced 2‐AG suggests potential ECS involvement in thermal‐stress adaptation. These findings provide a foundation for future research on nutritional or management strategies aimed at improving resilience to HS.

## INTRODUCTION

Heat stress (HS), characterised by an environmentally induced increase in body temperature above the physiological range, is a major factor limiting livestock fertility in tropical areas.^1^ The dramatic changes in global climate, with more than 1.5°C increase in air temperature recorded from the preindustrial period^2^ make HS one of the major stressors affecting productive and reproductive performance of dairy cows. Temperature‒humidity index (THI) serves as a critical environmental metric for assessing HS in cattle.^3^, ^4^, ^5^ A THI value of 72 or higher is widely recognised as the threshold at which thermal stress begins to adversely affect the physiological and productive performance of dairy cows.^6^ A negative correlation has been reported between elevated body temperature, fertility and milk production in lactating dairy cows.^3^ The reduction in fertility induced by HS involves alterations in follicular growth, oocyte competence, ovulation, corpus luteum function and embryo development.^7^, ^8^, ^9^, ^10^ Moreover, alterations in the endometrium and uterine environment that may reduce fertility have also been described.^11^ Heat stress activates the hypothalamic‒pituitary‒adrenal (HPA) axis and increases peripheral levels of glucocorticoids.^12^, ^13^ The cortisol released during chronic stress causes immune suppression^14^ by inhibiting the synthesis of cytokines such as interleukin‐4 (IL‐4), IL‐5, IL‐6, IL‐12, interferon‐γ and tumour necrosis factor‐alpha (TNF‐α), resulting in a higher susceptibility to disease and immune challenges.^15^ Furthermore, HS affects fertility because the increased cellular metabolism leads to oxidative stress.^16^ It is known that the redox status of the oocytes is compromised due to increased reactive oxygen species (ROS) production, reduced glutathione levels and antioxidant enzymatic activity.^2^ These physiological, endocrine and immunological alterations induced by HS highlight the need to explore additional regulatory mechanisms involved in stress adaptation, among which the endocannabinoid system (ECS) has recently gained increasing attention.

The most studied endocannabinoids (eCBs), N‐arachidonylethanolamide (anandamide, AEA) and 2‐arachidonoylglycerol (2‐AG), are involved in the regulation of the HPA axis.^17^ Pharmacological enhancement of eCB signalling has been shown to attenuate the stress response by reducing corticosterone release.^18^ Moreover, AEA and 2‐AG have complex immunomodulatory effects, exhibiting both anti‐ and pro‐inflammatory properties depending on the tissue and cell type; for example, AEA suppresses cytokine production in T lymphocytes, whereas 2‐AG can promote pro‐inflammatory signalling in neutrophils and monocytes.^19^ These effects are mediated in part through the cannabinoid receptor type 2 (CB2), which is highly expressed on immune cells, including macrophages, lymphocytes and neutrophils.^20^ Cannabinoid receptor type 2 activation generally suppresses pro‐inflammatory cytokine release, inhibits immune cell migration and promotes regulatory phenotypes in macrophages. This contributes to the resolution of inflammation and maintenance of immune homeostasis.^21^ In addition to anti‐inflammatory activity, AEA and 2‐AG can be metabolised into arachidonic acid derivatives, which may have pro‐inflammatory effects, and their impact can vary with concentration, receptor expression and the specific immune cell involved.^20^ Endocannabinoid system can also be affected by environmental heat load, as decreased expression of ECS genes was detected in the adipose tissue of cows during summer versus winter.^22^

Given this background, the present study aimed to evaluate the oxidative status, inflammatory responses and ECS activity in dairy cows during winter and summer seasons. This study focused on clarifying the involvement of these pathways in response to HS.

## MATERIALS AND METHODS

### Animals

The study included 60 Italian Holstein dairy cows. The cows were recruited from three farms located in different regions of southern Italy. Specifically, the first farm was situated in the rural area of Caserta, Campania; the second in the rural area of Messina, Sicily; and the third in the rural area of Bari, Apulia. A total of 10 cows were enrolled from each farm per season.

The animals were housed in a free‐stall system and were fed a total mixed ratio based on oat hay, alfalfa hay, alfalfa meal, crushed barley and beet pulp. The three farms were characterised by comparable management practices, including similar feeding strategies and routine herd management protocols.

All enrolled animals were between 2 and 5 years old, in their first to third lactation, with an average body condition score (BCS) of 3, and were between 60 and 80 days in milk.

### Temperature‒humidity index evaluation

Ambient temperature (AT) and relative humidity (RH) were continuously monitored for 1 month before sample collection using a data logger placed in the feeding area of the animals. The device was positioned at the approximate head height of the animals to ensure accurate representation of the environmental conditions they were directly exposed to. Based on temperature and humidity values, the THI was calculated using the following established formula widely used and validated for evaluating HS in dairy cattle under field conditions and other species, allowing comparison with previously published studies and established thermal stress thresholds.^23^, ^24^

THI = (1.8 × AT + 32) − (0.55 − 0.0055 × RH) × [(1.8 × AT + 32) − 58]

Here, AT is expressed in degrees Celsius, which is converted to degrees Fahrenheit by the expression: 1.8 × AT + 32, and RH is expressed as a decimal fraction.

A THI value greater than or equal to 72 has been considered the threshold for the onset of thermal stress in dairy cows.^6^

### Study design

The study was conducted in January 2024 for the winter season and in July 2024 for the summer season. Before any experimental procedures, all cows underwent a thorough clinical examination, including rectal exploration, to rule out any potential pathologies. Only animals without concurrent diseases were included in the study.

At the time of sampling, all the enrolled animals were distributed into three groups of 10 cows each: groups Caserta, Messina and Bari.

At enrolment, the three groups were comparable for age, lactation number, BCS and days in milk distribution. Oestrus detection was performed using pedometers and confirmed by clinical examination. The pedometers, connected to herd management software, monitored each animal's movement. A sharp increase in the number of daily steps indicated the estrus phase. After detecting the increase in daily steps, the cows underwent rectal palpation to assess uterine tone typical of heat and the presence of preovulatory follicles at the ovarian level. Ultrasonographic examination confirmed the presence of Graafian follicles based on their size (1.9–2 cm).

Blood was collected from the coccygeal vein using serum Vacutainer tubes without a gel separator. To avoid possible influences of circadian rhythm and feeding on the tested parameters, all samples were collected between 7:00 and 8:00 am before feeding. All samples were transported immediately to the reference laboratory (20 ± 10 min) and centrifuged for 10 min at 3000 rpm at +4°C. The obtained serum was then aliquoted into 2 mL Eppendorf tubes and stored at ‒20°C until analysis.

### Laboratory analysis

#### Oxidative status analysis

The serum aliquots used for the determination of biological antioxidant potential (BAP) and reactive oxygen metabolite (d‐ROMs) concentrations were transferred to the Department of Veterinary Medicine of the University of Bari ‘Aldo Moro’. All analyses were conducted in the laboratory of the Obstetrics Clinic Section.

Biological antioxidant potential serum level was determined using the BAP test (Diacron International srl) through the FREE Carpe Diem photometric system (Diacron International srl). This test evaluates the biologically active antioxidant capacity of the blood barrier, which can reduce an oxidative substrate. The results are expressed in micromoles of reduced iron per litre of serum sample.

The concentrations of d‐ROMs were determined using the d‐ROM test (Diacron International srl) with the FREE Carpe Diem system (Diacron International srl). This photometric assay measures the oxidation induced by free radicals derived from hydroperoxides present in the sample. The results of this test are expressed in arbitrary units, defined as Carratelli units (U.CARR.), where 1 U.CARR. corresponds to 0.08 mg H_2_O_2_/dL.

#### Inflammatory status analysis

The inflammatory status of the animals enrolled in this study was also assessed by the Endocannabinoid Research Group at the Institute of Biomolecular Chemistry of the National Research Council, Pozzuoli, Naples, Italy. Analyses were performed to measure serum levels of TNF‐α and IL‐10 by SinglePlex ELISA kit (Cusabio) with a GENios‐Pro Reader (Tecan) following the manufacturer's instructions.^25^

The detection ranges were 0.1‒20 ng/mL and 5‒1000 pg/mL for TNF‐α and IL‐10, respectively. For sensitivity, the minimum detectable dose of bovine TNF‐α was less than 0.05 ng/mL and that of IL‐10 was 2.5 pg/mL. The assays have high sensitivity and specificity for detection of these bovine cytokines.

#### Endocannabinoid analysis

The serum aliquots used for eCB assays were shipped to the Institute of Biomolecular Chemistry of the National Research Council, Pozzuoli, Naples, Italy. To ensure proper preservation of the samples during transport, they were placed inside an isothermal container and covered with dry ice. The measurements of the eCBs were performed by the Endocannabinoid Research Group. The eCBs were analysed and quantified after the extraction and purification procedures, within 2 months of sample collection. Serum samples were extracted in 5 volumes of chloroform/methanol (2:1), containing 5 pmol of arachidonoyl ethanolamide‐d8 (d8‐AEA) and 2‐arachidonoyl glycerol‐d5 (d5‐2‐AG) (Cayman Chemicals). The lipid‐containing organic phase was dried down in a rotating evaporator. It was then pre‐purified by open‐bed chromatography on silica gel columns eluted with increasing concentrations of methanol in chloroform. Fractions eluted with chloroform/methanol (9:1 by volume) (containing AEA and 2‐AG) were collected and aliquots were analysed by isotope dilution liquid chromatography/atmospheric pressure chemical ionisation/mass spectrometry (LC–APCI–MS) using a Shimadzu high‐performance liquid chromatography apparatus (LC‐10ADVP) coupled to a Shimadzu quadrupole mass spectrometer (LCMS‐2020) via a Shimadzu APCI interface. Liquid chromatography analysis was performed in the isocratic mode using a Kinetex C18 column (Phenomenex, 15 cm × 4.6 mm, 5 µm) and methanol/water/acetic acid (85:15:1 by volume) as mobile phase with a flow rate of 1 mL/min. Mass spectrometry detection was carried out in the selected ion monitoring mode using m/z values of 356 and 348 (molecular ion +1 for deuterated and undeuterated AEA) and 384.35 and 379.35 (molecular ion +1 for deuterated and undeuterated 2‐AG). The levels of eCBs were then calculated based on their area ratios with the internal deuterated standard signal areas, and their amounts were expressed as pmol/mL of serum.

### Statistical analysis

All the data were collected in an Excel spreadsheet and statistical analyses were performed using RStudio version 2025.05.1. All the data are expressed as mean ± standard deviation. The distribution of each variable (THI, BAP, d‐ROMs, TNF‐α, IL‐10, AEA and 2‐AG) was assessed for normality using the Shapiro‒Wilk test, which was applied both to the entire dataset and separately within each season (‘winter’ and ‘summer’).

Based on the results of the normality tests, the homogeneity of variances was evaluated using either Bartlett's test (for normally distributed data) or Levene's test (for non‐normally distributed data).

Depending on the distribution and variance homogeneity, comparisons of parameters among the three different farms within each season were performed using either a one‐way analysis of variance (ANOVA) or the Kruskal‒Wallis test. When significant differences were detected, appropriate post hoc analyses were conducted using Tukey's honestly significant difference test following ANOVA, or the Dunn test following the Kruskal‒Wallis test, to identify specific pairwise differences between farms.

For comparisons of overall parameters between the two seasons (‘winter’ vs. ‘summer’), either the Student's *t*‐test or the Wilcoxon–Mann–Whitney *U*‐test was applied, according to the assumptions of normality and variance homogeneity.

Correlations between variables were assessed using Spearman's rank correlation coefficient (*ρ*). The strength and direction of associations were interpreted according to the sign and magnitude of *ρ*, with positive values indicating a direct correlation and negative values indicating an inverse correlation. Correlation strength was categorised as weak (*ρ* < 0.3), moderate (0.3 ≤ *ρ* < 0.7) or strong (*ρ* ≥ 0.7). Corresponding *p*‐values were computed to evaluate the statistical significance of the correlations.

A significance level of a *p*‐value of less than 0.05 was considered statistically significant for all analyses.

## RESULTS

As shown in Table 1, the data obtained in this study did not reveal any statistical differences among farms within individual seasons for any of the considered variables. Therefore, the differences between seasons were considered for each measured parameter by taking the average of the three farms within the season into account.

**TABLE 1:** Mean values (± standard deviation) of temperature‒humidity index (THI), biological antioxidant potential (BAP), reactive oxygen metabolites (d‐ROMs), tumour necrosis factor‐alpha (TNF‐α), interleukin‐10 (IL‐10), anandamide (AEA) and 2‐arachidonoylglycerol (2‐AG) in dairy cows from the three farms (Caserta, Messina and Bari) during the summer and winter seasons.

	Summer			Winter		
	Caserta	Messina	Bari	Caserta	Messina	Bari
THI	74.31 ± 1.04	76.57 ± 1.75	76 ± 1.04	58.97 ± 0.56	57.70 ± 0.90	57.75 ± 0.95
BAP (µmol/L)	3187.25 ± 383.90	2960.81 ± 314.67	3164.28 ± 239.82	3350.74 ± 444.14	3226.7 ± 203.27	3327.47 ± 282.19
d‐ROMs (U.CARR.)	66.85 ± 8.35	67.21 ± 7.45	68.72 ± 10.86	56.01 ± 6.96	56.09 ± 11.45	58.93 ± 11.06
TNF‐α (ng/mL)	1.58 ± 0.30	1.53 ± 0.21	1.51 ± 0.25	1.33 ± 0.22	1.33 ± 0.21	1.32 ± 0.21
IL‐10 (pg/mL)	51.08 ± 11.01	54.75 ± 18.83	49.37 ± 9.26	49.38 ± 11.68	40.83 ± 8.05	47.75 ± 13.12
AEA (pmol/mL)	1.94 ± 1.15	2.07 ± 0.73	1.84 ± 0.66	2.01 ± 1.27	2.12 ± 0.51	2.47 ± 0.97
2‐AG (pmol/mL)	18.45 ± 12.83	17.78 ± 6.97	14.09 ± 3.47	21.24 ± 9.67	26.59 ± 8.71	21.31 ± 10.98

### Temperature‒humidity index

**FIGURE 1:**
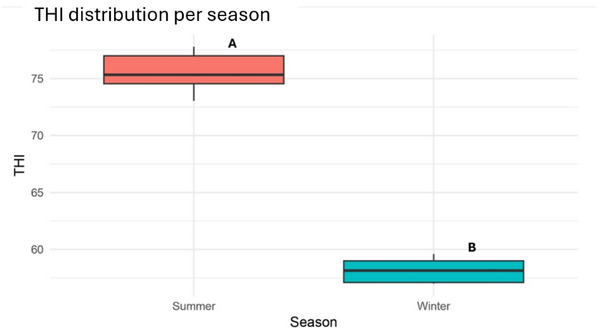
Temperature‒humidity index (THI) mean value distribution in dairy farms during summer and winter seasons. Different letters (A and B) indicate statistically significant differences between seasons (*p* < 0.001).

Significant differences were observed for THI between seasons, with considerably higher values recorded in summer (75.63 ± 1.56) compared to winter (58.14 ± 0.96, *p* < 0.001), as shown in Figure 1.

### Oxidative status

**FIGURE 2:**
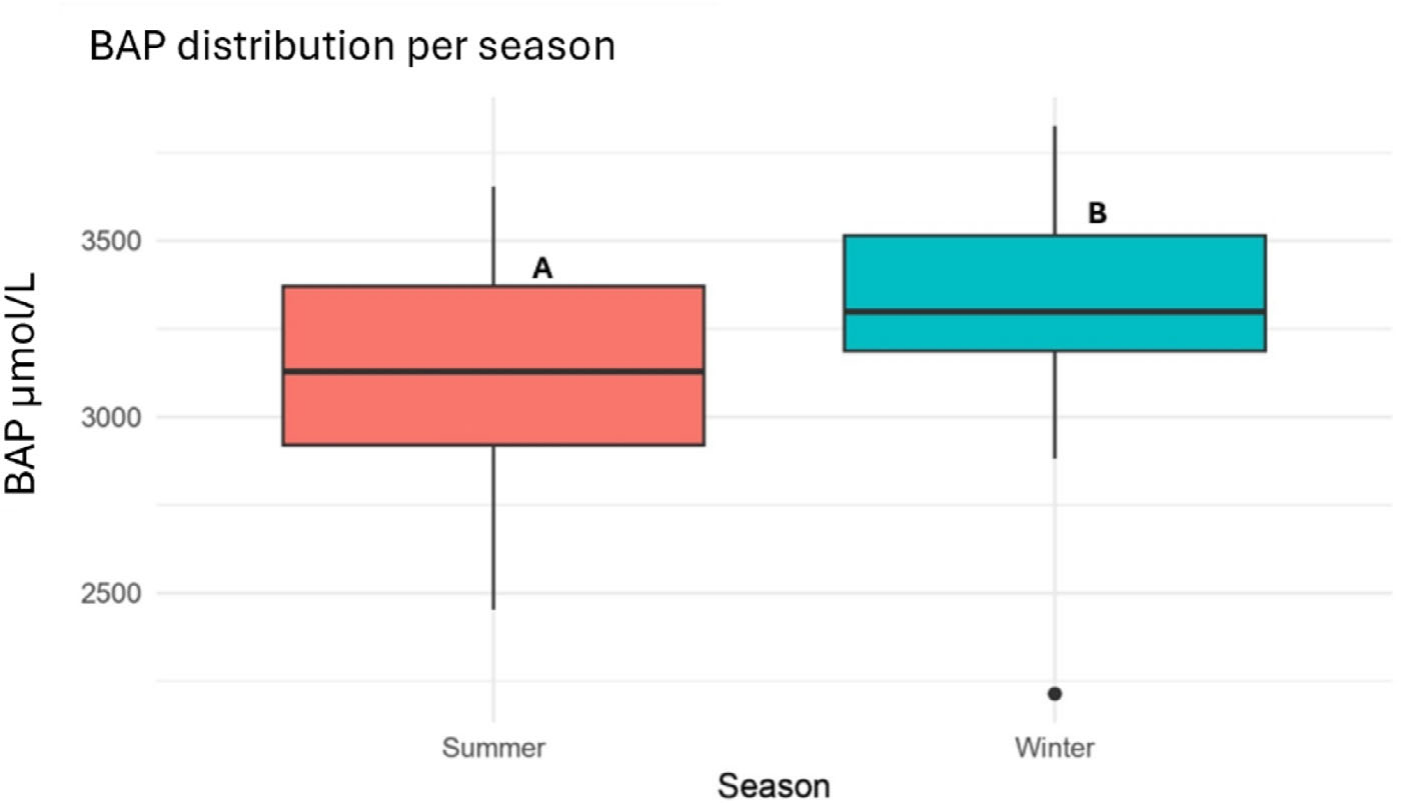
Biological antioxidant potential (BAP) values (µmol/L) distribution in dairy farms during summer and winter seasons. Different letters (A and B) indicate statistically significant differences between seasons (*p* < 0.05).

**FIGURE 3:**
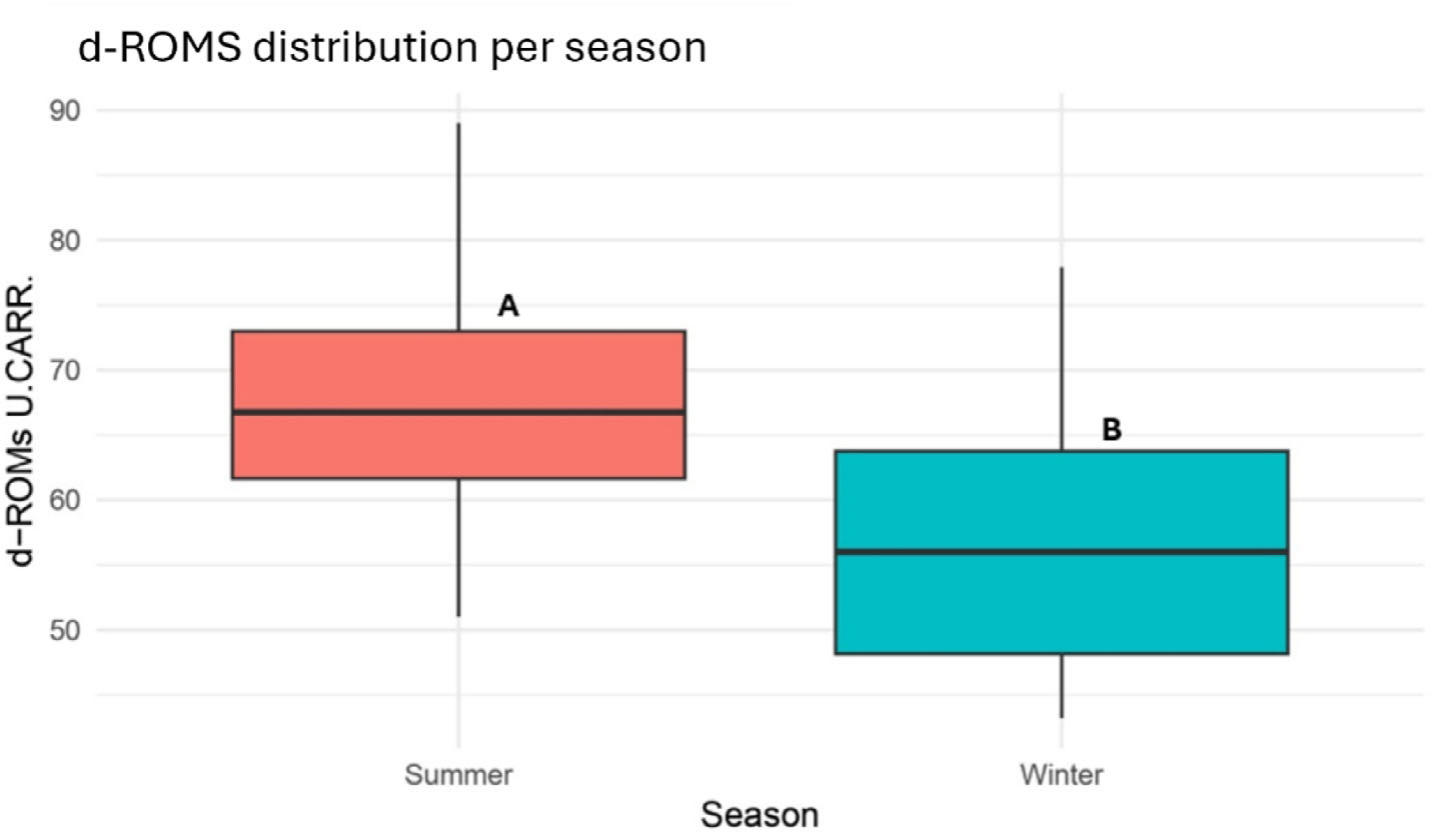
Reactive oxygen metabolites (d‐ROMs) concentrations (U.CARR.) distribution in dairy farms during summer and winter seasons. Different letters (A and B) indicate statistically significant differences between seasons (*p* < 0.001).

The biological antioxidant potential levels were significantly lower in summer (3104.11 ± 324.08 µmol/L) compared to winter (3301.64 ± 318.99 µmol/L, *p <* 0.05) (Figure 2). In contrast, d‐ROM concentrations increased significantly from 57.01 ± 9.78 U.CARR. in winter to 67.59 ± 8.73 U.CARR. in summer (*p <* 0.001) (Figure 3).

### Inflammatory status

**FIGURE 4:**
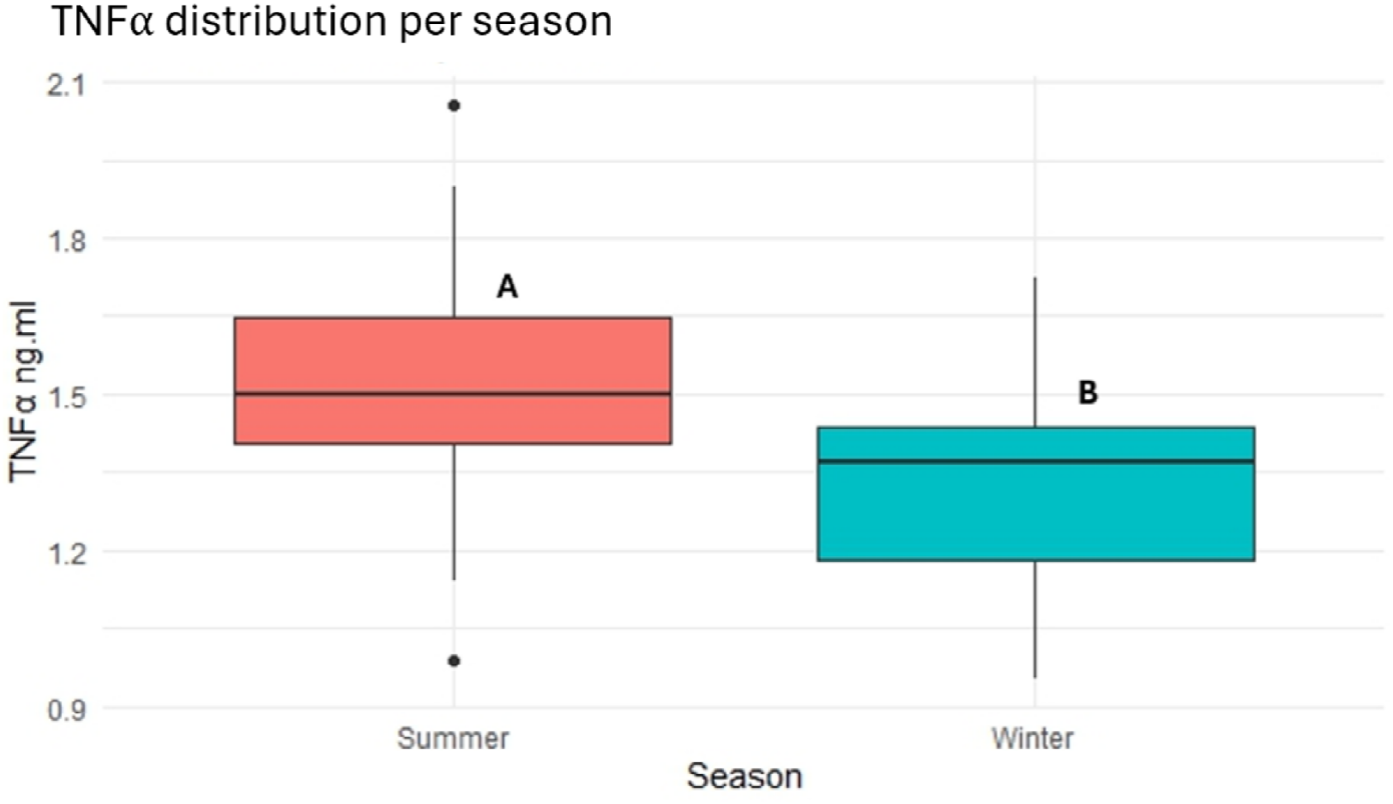
Tumour necrosis factor‐alpha (TNF‐α, ng/mL) distribution in dairy farms during summer and winter seasons. Different letters (A and B) indicate statistically significant differences between seasons (*p* < 0.001).

However, TNF‐α levels increased in summer compared to winter (1.54 ± 0.24 vs. 1.33 ± 0.20 ng/mL, *p <* 0.001), as shown in Figure 4.

**FIGURE 5:**
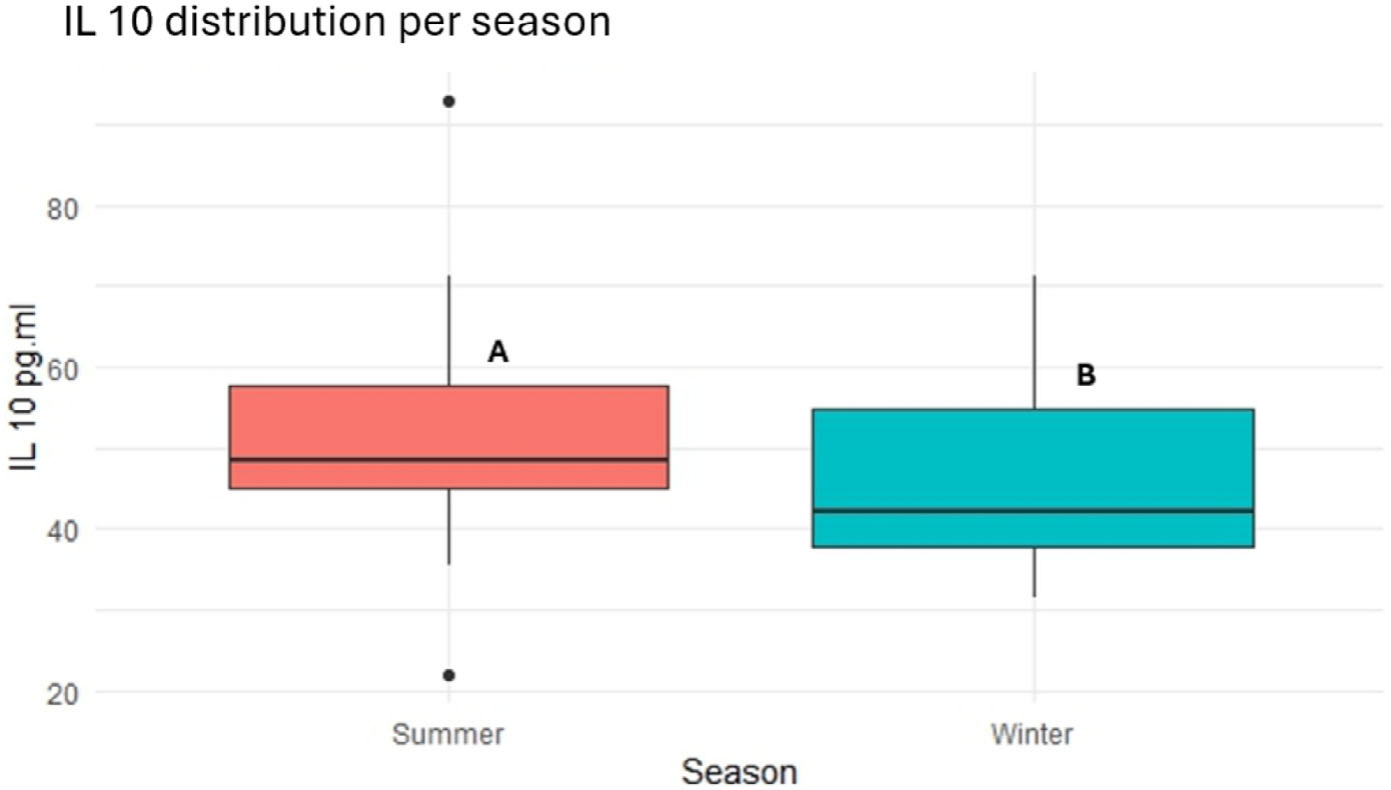
Interleukin‐10 (IL‐10, pg/mL) distribution in dairy farms during summer and winter seasons. Different letters (A and B) indicate statistically significant differences between seasons (*p* < 0.05).

Furthermore, IL‐10 levels were higher in summer than in winter (51.73 ± 13.40 vs. 46.10 ± 11.40 pg/mL, *p* *<* 0.05, as depicted in Figure 5.

### Endocannabinoid levels

**FIGURE 6:**
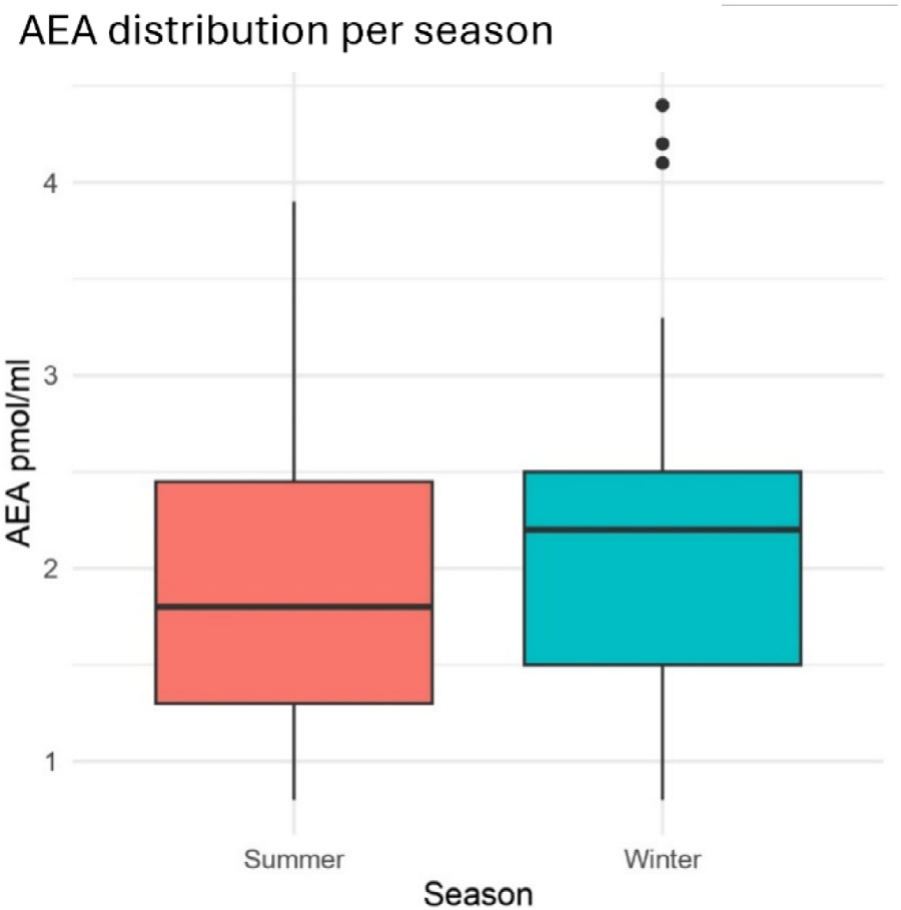
Anandamide (AEA) concentration (pmol/mL) distribution in dairy farms during summer and winter seasons.

No differences in AEA concentrations were detected between seasons (1.95 ± 0.85 and 2.20 ± 0.96 pmol/mL, respectively in summer and winter), as shown in Figure 6.

**FIGURE 7:**
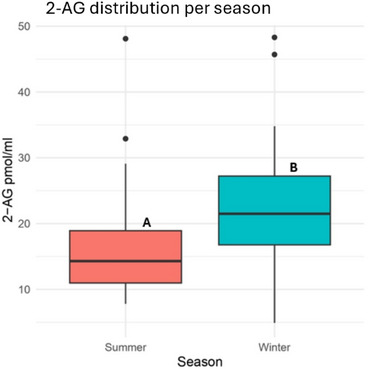
2‐Arachidonoylglycerol (2‐AG) concentration (pmol/mL) distribution in dairy farms during summer and winter seasons. Different letters (A and B) indicate statistically significant differences between seasons (*p* < 0.01).

Regarding 2‐AG concentrations, a significant seasonal decline was detected from winter (23.05 ± 9.82 pmol/mL) to summer (16.77 ± 8.59 pmol/mL, *p* < 0.01), as illustrated in Figure 7.

### Variables correlation

**FIGURE 8:**
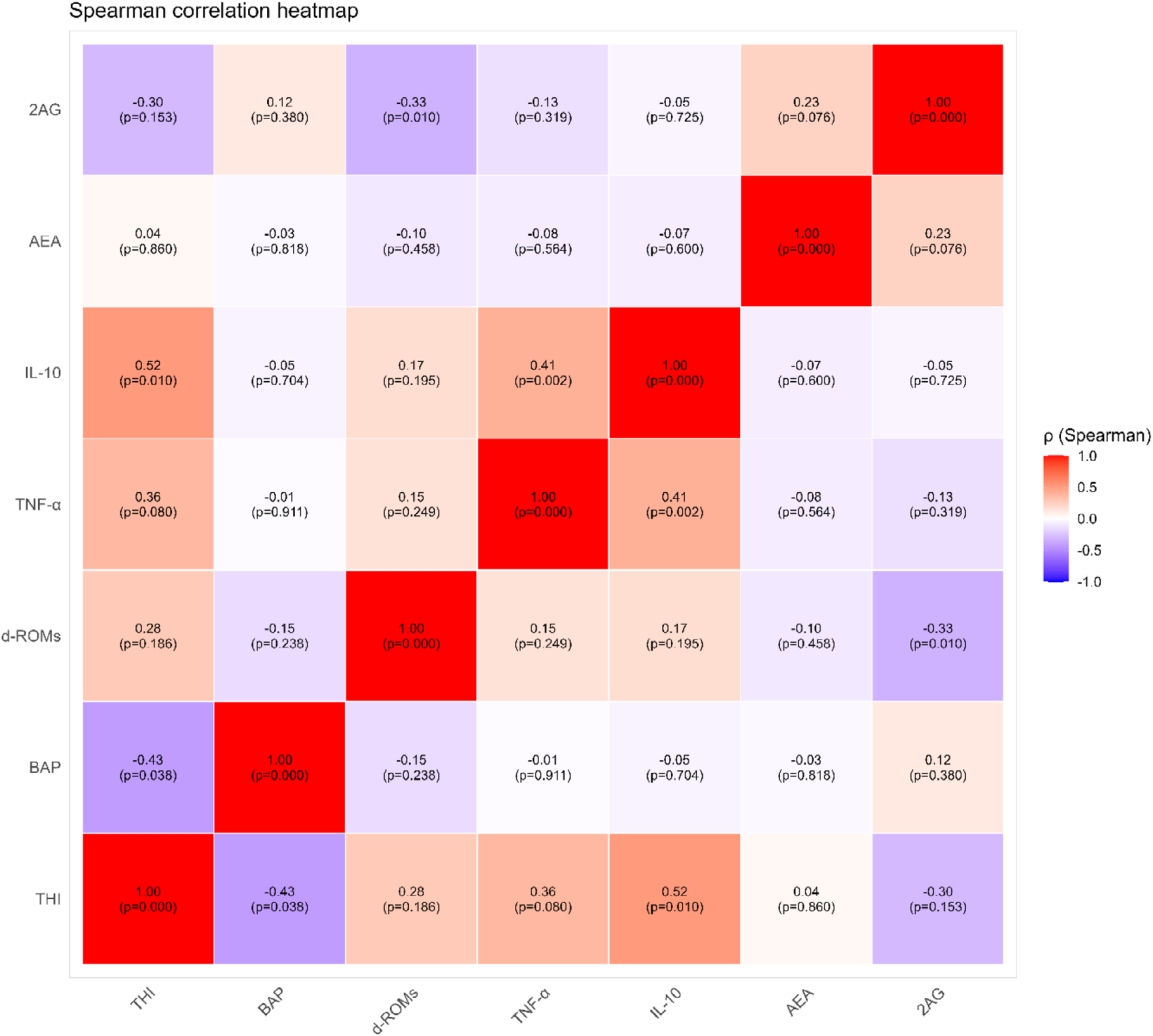
Correlation analysis represented using Spearman's rank correlation coefficients (*ρ*) with statistical significance indicated by the corresponding *p*‐values between the measured variables: temperature‒humidity index (THI), biological antioxidant potential (BAP), reactive oxygen metabolites (d‐ROMs), tumour necrosis factor‐alpha (TNF‐α), interleukin‐10 (IL‐10), anandamide (AEA) and 2‐arachidonoylglycerol (2‐AG).

Correlation analysis identified some significant associations among the measured variables (Figure 8). Temperature‒humidity index was moderately negatively correlated with BAP (*ρ* = −0.43, *p =* 0.038) and moderately positively correlated with IL‐10 (*ρ* = 0.52, *p =* 0.010). A moderate negative correlation was detected between d‐ROMs and 2‐AG (*ρ* = −0.33, *p =* 0.010). In addition, a moderately positive correlation was found between TNF‐α and IL‐10 (*ρ* = 0.41, *p =* 0.0016).

## DISCUSSION

In recent years, the need to develop effective strategies to control and mitigate HS in dairy cattle farms has grown considerably.^26^ Numerous studies have already demonstrated the detrimental effects of this condition on overall health and fertility in dairy cows.^27^, ^28^, ^29^, ^30^ In this context, oxidative status, inflammatory profile and the ECS are endogenous systems particularly sensitive to HS and thus, were the focus of the present study. Oestrus was used as a methodological condition to standardise reproductive status across animals and reduce physiological variability unrelated to HS.

The first step of the study involved calculating the THI in three dairy farms located in different Italian regions (Campania, Sicily and Apulia), both during the winter and summer seasons. No significant differences emerged among the farms within the same season. As expected, average summer THI values were significantly higher than those recorded in winter, exceeding the critical threshold of 72, above which dairy cows are known to exhibit physiological and behavioural signs of HS.^31^

Prolonged exposure to elevated THI values has been shown to reduce feed intake, cause endocrine alterations (such as increased cortisol and adrenocorticotropic hormone levels), and impair thermoregulation, directly affecting fertility and productivity.^32^ Moreover, heat‐induced cellular damage can activate oxidative and pro‐inflammatory pathways, further worsening the animals’ general health condition.^15^

The oxidative profile observed under summer conditions is consistent with previous studies demonstrating that HS promotes oxidative stress through enhanced ROS production and reduced antioxidant capacity, compromising the animal's ability to counteract ROS‐induced cellular damage.^33^, ^34^

Higher levels of both cytokines were observed during the summer compared to the winter season in agreement with previous findings.^35^ The observed concentrations were consistent to those reported in other studies.^35^, ^36^, ^37^ Considering the high susceptibility of cytokine expression to physiological and environmental factors, the observed seasonal increase likely reflects the pro‐inflammatory effect of HS.^38^, ^39^, ^40^

The ECS was evaluated in this study by measuring the serum concentrations of its two main compounds, AEA and 2‐AG. The results showed that both AEA and 2‐AG tended to decrease during the summer compared to winter, although the reduction reached statistical significance only for 2‐AG.

Exposure to high environmental temperatures promotes a systemic inflammatory state in dairy cows,^22^ associated with increased expression and activity of the enzyme cyclooxygenase‐2 (COX‐2), a key mediator in the biosynthesis of pro‐inflammatory prostaglandins and thermoregulatory processes.^38^ Cyclooxygenase‐2 is also involved in the metabolism of eCBs, particularly AEA and 2‐AG, which may be degraded by this enzyme.^41^ The oxidation of these eCBs leads to the formation of prostaglandin‐ethanolamides and prostaglandin‐glycerols, respectively.^41^, ^42^ Based on previous evidences, this mechanism could hypothetically explain the observed decrease in serum 2‐AG concentrations during the summer, and the tendency towards reduced AEA levels, although not statistically significant.

In addition, chronic HS has been reported to enhance the expression and activity of fatty acid amide hydrolase, the enzyme responsible for AEA hydrolysis into arachidonic acid and ethanolamine,^42^ potentially contributing to further AEA reduction.

The reduced levels of 2‐AG, which is known to have an anti‐inflammatory and modulatory effect on cytokine production, is consistent with the idea that animals under seasonal HS are subjected to chronic inflammatory status.^19^

Correlation analysis provided further insights into the relationships between thermal stress, oxidative status, inflammatory markers and ECS in dairy cows. The negative association observed between THI and BAP could confirm that HS compromises redox balance by depleting circulating antioxidants and enhancing free radical production in dairy cattle during summer conditions.^33^ The concomitant positive correlation between THI and IL‐10 may reflect the activation of compensatory anti‐inflammatory responses, which are frequently described in heat‐stressed ruminants as part of the systemic adaptation to thermal load and oxidative injury.^38^

The negative correlation between d‐ROMs and 2‐AG observed in the present study could suggest a possible link between oxidative stress and eCB metabolism in dairy cows exposed to HS, associated with induction of COX‐2.^43^ Finally, the positive correlation between TNF‐α and IL‐10 highlights a concomitant regulation of pro‐ and anti‐inflammatory cytokines, reflecting their reciprocal involvement in the activation of immune processes.^35^ In particular, as reported in the literature, it seems that IL‐10 increases in order to counterbalance the rise in TNF‐α levels.^36^

To our knowledge, this study provides novel evidence on the potential involvement of the ECS in the physiological response to HS in dairy cows, suggesting a possible interaction with oxidative status and inflammatory cytokines. The concomitant evaluation of these systems suggests a complex and integrated response to HS. It is important to note that the data reported in this study refer exclusively to Italian Holstein Friesian cows. Consequently, these findings should not be generalise to other breeds that exhibit higher thermal tolerance, as has been demonstrated in other studies.^44^ However, it is also important to consider that, although several confounding factors were partially controlled through the study design, additional animal‐ and farm‐level variables were not included in statistical analysis.

### Study limitations

Moreover, the present investigation was exploratory in nature. Although the sample size of 30 animals per season was sufficient to detect significant seasonal differences in key parameters, such as 2‐AG, it was not determined by a priori power calculation. This may limit the ability to detect smaller effect sizes and may also introduce potential confounding effects due to unaccounted variables; therefore, further investigations with a larger sample size and inclusion of additional controlled variables are recommended to strengthen and extend the present evidence.

## CONCLUSIONS

The results of this study indicated that cows subjected to HS experienced alterations in their oxidative status, inflammatory profile and ECS. During the summer season, high THI values were associated with reduced antioxidant capacity and increased oxidant production. Similarly, there was an increase in inflammatory cytokine levels, confirming that HS could induce chronic inflammation. Additionally, ECS exhibited reduced levels of eCBs, potentially due to an increase in enzymes capable of degrading them in response to HS.

Considering these findings, a promising starting point for future research involves evaluating the effectiveness of targeted nutritional strategies capable of positively modulating the ECS and enhancing the antioxidant and anti‐inflammatory capacities of dairy cows. Such interventions could represent a promising approach not only to improve animal welfare but also to support productive performance in cows chronically exposed to HS conditions.

## AUTHOR CONTRIBUTIONS

*I* *nvestigation, methodology, data curation, formal analysis, writing—original draft and writing—review and editing*: Alice Carbonari. *Investigation, methodology, data curation, formal analysis, writing—original draft and writing—review and editing*: Lorenza Frattina. *Methodology and writing—review and editing*: Matteo Burgio, Vincenzo Cicirelli, Michal Andrzej Kosior and Alessandra Sfacteria. *conceptualization, methodology and writing—review and editing*: Bianca Gasparrini and Gabriele Marino. *Methodology, investigation and writing—review and editing*: Roberta Verde and Fabiana Piscitelli. *Investigation, conceptualization, methodology, writing—review and editing and supervision*: Annalisa Rizzo.

## CONFLICTS OF INTEREST

The authors declare they have no conflicts of interest.

## ETHICS STATEMENT

All procedures were carried out in accordance with institutional animal welfare guidelines, with prior informed consent obtained from the owners. Ethical approval was granted by the Ethics Committee of the Department of Veterinary Medicine, University of Bari ‘Aldo Moro’, under protocol number no. 31/2023.

## Data Availability

The data presented in this study are available upon request from the corresponding author.
